# Six-month adherence to Statin use and subsequent risk of major adverse cardiovascular events (MACE) in patients discharged with acute coronary syndromes

**DOI:** 10.1186/s12944-017-0544-0

**Published:** 2017-08-15

**Authors:** Gaoqiang Xie, Yihong Sun, Phyo Kyaw Myint, Anushka Patel, Xingzi Yang, Min Li, Xian Li, Tao Wu, Shenshen Li, Runlin Gao, Yangfeng Wu

**Affiliations:** 1 Peking University Clinical Research Institute, Beijing, China; 2 Key Laboratory of Molecular Cardiovascular Sciences, Ministry of Education, Beijing, China; 3 China-Japan Friendship Hospital, Beijing, China; 4 School of Medicine, Medical Sciences and Nutrition, University of Aberdeen, Aberdeen, UK; 5 The George Institute for Global Health, University of Sydney, Sydney, Australia; 6 Department of Epidemiology and Biostatistics, |Peking University School of Public Health, Beijing, China; 7 The George Institute for Global Health at Peking University Health Science Center, Beijing, China; 8 The Department of Cardiology, Fuwai Hospital, National Center for Cardiovascular Diseases, Chinese Academy of Medical Sciences, Beijing, China

**Keywords:** Adherence, Statin, Major adverse cardiovascular events (MACEs), Acute coronary syndrome (ACS), Cohort

## Abstract

**Background:**

The evidence of adherence to statin decreasing risk of major adverse cardiovascular events (MACEs) is still lack among patients discharged with acute coronary syndrome (ACS).

Our objective is to determine the relationship between six-month adherence to statins and subsequent risk of MACEs in patients discharged with ACS.

**Methods:**

Using two prospective registry cohorts (CPACS-1 and -2), we analyzed data from 12,516 consecutive patients with ACS who were prescribed statin at hospital discharge and survived beyond 6 months without recurrent myocardial infarction (MI) or stroke. Adherence to statin was defined as good (using statin at discharge and 6 months without declined dosage) and poor adherence groups (using statin at discharge but declining dosage or stopping at 6 months). We compared the hazard ratios of all-cause mortality and MACE in subsequent 6 months between groups, using Cox-regression models, adjusting for multiple potential confounders.

**Results:**

Seventy two percent of patients adhered to statin therapy at 6 months. The incident MACE in the poor adherence group was significantly higher than in good adherence group (2.7% vs. 1.8%, *p* = 0.002). Compared with poor adherence group, the good adherence group showed a 27% lower relative risk of MACE during the 6 month follow up (fully-adjusted hazard ratio (HR) = 0.73; 95%CI: 0.56–0.97). The protective effects of good adherence were similar in groups with different statin dose as well as groups by other baseline clinical characteristics and treatments (*p* > 0.05 for interaction).

**Conclusion:**

Our study highlights the importance of adherence to statin therapy in prevention of MACE and clinicians should aim to achieve higher dosage if tolerable.

**Clinical trial registration:**

CPACS2 was registered on URL: http://www.anzctr.org.au/default.aspx and unique identifier is ACTRN12609000491268. CPACS1 was not a clinical trial and thus not registered.

**Electronic supplementary material:**

The online version of this article (doi:10.1186/s12944-017-0544-0) contains supplementary material, which is available to authorized users.

## Background

It was estimated that 8.1 million Chinese were affected by coronary heart disease (CHD) in 2010 [1]. The CHD events are set to rise with projected increase by 69% between 2010 and 2030 in China [2]. Acute coronary syndrome (ACS) is the most serious presentation of CHD. Statins are the main stay of treatment in ACS both acutely and for secondary prevention. Long term use of statins reduces the subsequent risk of major adverse cardiovascular events (MACE) including mortality [3–6] when used in accordance with international clinical practice guidelines including Chinese guidelines [7–11].

Despite the strong evidence of protective effect of statins on subsequent cardiovascular risk, the uptake of statin therapy in China was modest after ACS; recent studies have indicated ~80% statin prescription at discharge which is compounded by subsequent attrition to adherence rates of less than 60% by 12 months of follow-up [12, 13].

Medication adherence is defined as the extent to which a patient takes medications as prescribed by their healthcare providers [14]. Poor adherence reduces the effectiveness of essential medications and has been highlighted as a significant obstacle in achieving good patient outcomes [14]. The benefit of adherence to statin on MACE has been confirmed in the primary prevention of coronary artery disease among healthy populations [15, 16]. However, studies on its effects in secondary prevention are still limited especially in patients discharged with ACS [17–19]. It is also unknown whether the effects are independent of good adherence to other evidence-based drugs therapy or not in these patients. Moreover, in Chinese ACS patients, about 67.1% did not have known dyslipidaemia [20] and about 42.8% used low-dose statin (<20 mg atorvastatin or equivalent) [13]. It is important to quantify the benefit of statin adherence in Chinese ACS patients with poor adherence, to provide incentive for greater adherence to statins for secondary prevention.

We therefore set out to determine the relationship between six-month self-reported adherence to statin use and subsequent risk of major adverse cardiovascular events (MACEs) in Chinese patients with acute coronary syndrome (ACS). We examined the relationship between six-month adherence to statin post ACS and MACE in the following 6 months among 12,516 ACS patients who had been prescribed to use statin at hospital discharge. We also aimed to assess if the relationship was independent of good adherence to other evidence-based beneficial drugs, and was consistent in groups with different statin dose as well as groups by other baseline clinical characteristics and treatments.

## Methods

### Study population

**Fig. 1:**
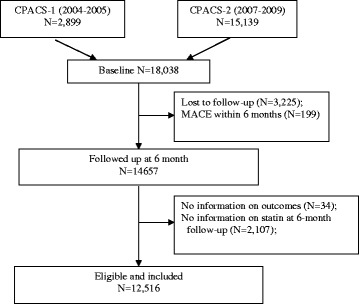
Flow chart of study participants

The study sample was drawn from two multicenter prospective registry studies of ACS. They are Clinical Pathways for Acute Coronary Syndromes in China (CPACS)-1 and-2 which were carried out between 2004 and 2005 and 2007–2009, respectively. The designs and previous findings of these registries have been reported elsewhere [12, 20–22]. The two studies designed by the same study group ensured that data on key variables such as statin usages (yes/no), death, MI, and stroke were collected with the same methods and definitions, providing the foundation to merge them for the current study. A total of 18,038 hospitalised patients with ACS were enrolled. These patients were then followed up at 6 and 12 months after discharge. In the present study, we analyzed 12,516 ACS patients who had been prescribed statins at discharge, survived to 6 months post-discharge without recurrent MI or stroke. These patients were then followed for further 6 months. The study schema is presented in Fig. 1.

### Statin data collection

Data on statin prescription at discharge and at 6 month after discharge were collected for each patient using standardized case report forms by trained staff in both cohorts. The commercial name, and period of using statin were collected. We defined the “good adherence” group as those using statin without decline of dosage between discharge and 6 months post discharge (*n* = 9019), and the “poor adherence” group as those using statin at discharge but with either cessation or dosage decline at 6 months post discharge (*n* = 3497). Dosage of statin was only collected in CPACS-2. In order to examine the interaction of statin dose on the relationship of statin adherence with MACE, we performed in subgroups by statin dose (5–10 mg/d and > = 20 mg/d equivalent dose of atorvastatin) using data from CPACS-2. For our analysis, the dosage of different statins was converted to the equivalent dosage of atorvastatin according to Chinese Guidelines on Prevention and Treatment of Dyslipidemia in Adults in 2007^7^ (Additional file 1: Table S1).

### Outcomes ascertainment

Data on outcomes including all-cause mortality, incident myocardial infarction (including fatal and non-fatal), and stroke (including fatal and non-fatal) were collected by two follow-up surveys (by clinic visit or telephone) at 6 and 12 months after discharge [12]. Major adverse cardiovascular event (MACE) was defined as composite end point of all-cause mortality, myocardial infarction, or stroke. All myocardial infarction and stroke events were diagnosed in hospitals by their physicians. In the present study, the outcome of interest was MACE occurring during 7–12 months after discharge and therefore, deaths, stroke or MI occurring within 6 months after discharge were excluded from the study.

### Other data collection

Other data collected during hospitalization included sociodemographic characteristics, medical history, features of the presenting condition, electrocardiographic (ECG) and plasma biomarker findings, as well as treatment received at the baseline and during hospitalization. Other data collected at discharge and 6-month follow-up included other prescribed medicines (aspirin, clopidogrel, beta-blocker, and angiotensin-converting enzyme inhibitor/angiotensin receptor blocker), medical history, presenting features, and diagnosis at discharge. These variables were analyzed as potential confounders in the present study.

### Statistical analyses

For descriptive purpose, means and standard deviations were calculated to present continuous variables and were compared by t-tests between poor and good adherence groups. Categorical variables were presented as number (percentage) and differences between groups were tested by Chi-squared analysis. Cox proportional hazards regression models (unadjusted, age- and sex- adjusted, and multiple co-variables-adjusted) were constructed to calculate hazard ratios (HR) and corresponding 95% confidence intervals (95%CI) for MACE occurring within 7–12 month post discharge. The co-variables that were adjusted in the models were selected based on the association metrics analyses and on the current professional knowledge. These co-variables included studies (CPACS-1,-2), subtypes of ACS, hospital level, health insurance, social economic status, clinical characteristics, concomitant medicines and reperfusion therapies during hospitalization, and adherence to other evidence-based secondary prevention treatments for ACS in the first 6 months, in addition to age and sex. All models were then also constructed for all-cause mortality, incident MI and stroke, separately.

Subgroup analyses were also performed with the Cox models in populations defined by age (< or > = 65 years), sex, type of ACS, presence of hypertension or, diabetes at baseline, smoking level, hospital type, baseline cardiovascular risk factors, coronary revascularization during hospital admission, dose of statin, adherence to other medications.

All *p* values reported were two sided, and a *p* value of < 0.05 was considered significant. All analyses were performed using SAS 9.4 (SAS Institute Inc., Cary, NC, USA).

## Results

### Baseline characteristics

**Fig. 2:**
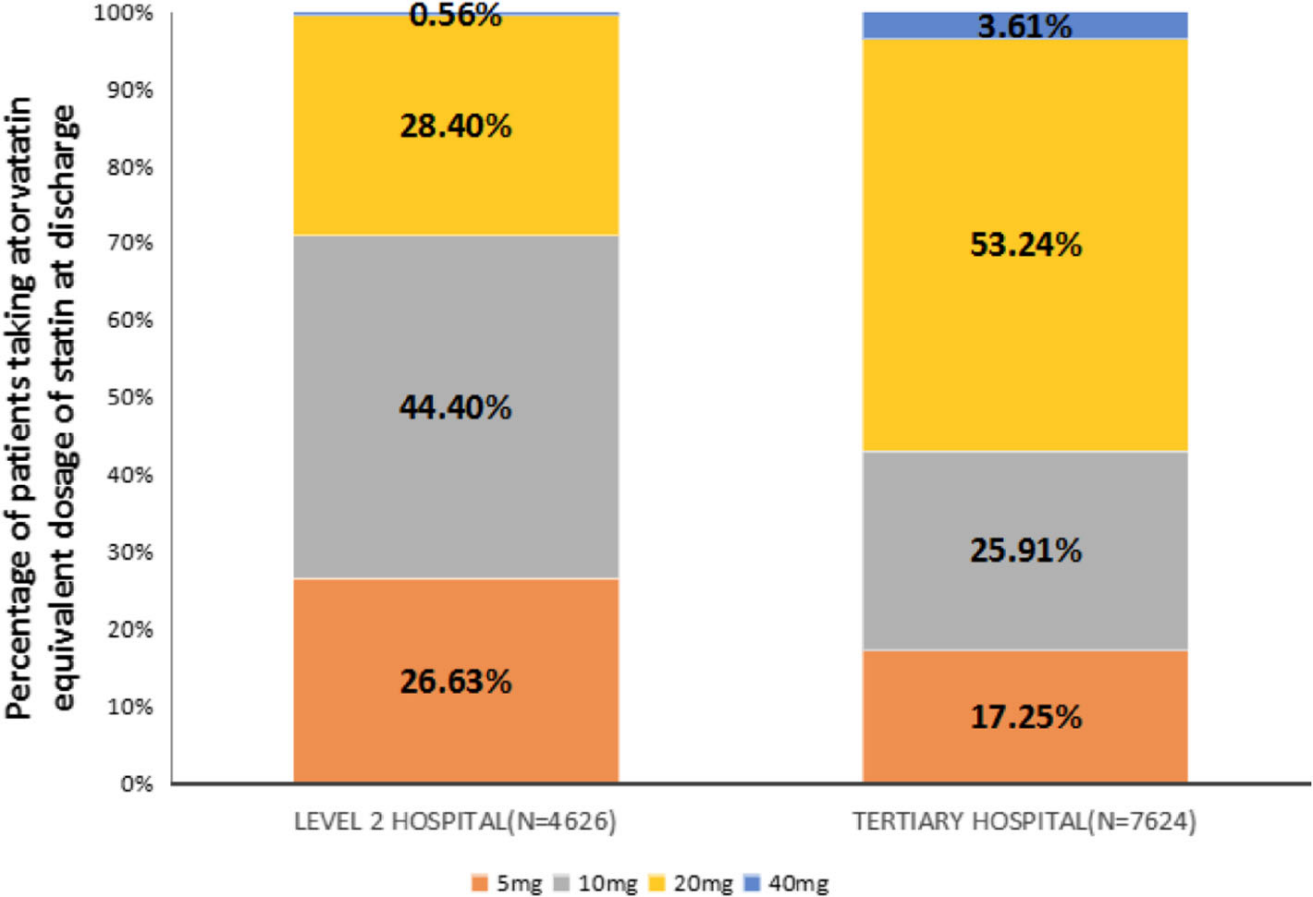
The proportion of patients prescribed different statin dosage in tertiary hospitals and level 2 hospitals (*p* < 0.001)

A total of the 12,516 patients (9019 men and 3497 women) were eligible to be included in this report (Fig. 1). The mean age (SD) of the cohort was 63.3 (11.5) years at the time of hospitalization. The rate of good adherence was 72% (9019/12516) at 6 months after discharge. Compared with the poor adherence group, the good adherence group had a higher proportion of men, and were more likely to have a higher education level, to be treated in tertiary hospital, to have health insurance, to have diabetes mellitus, dyslipidemia, and taking statins before hospitalization. During hospitalization, the good adherence group were more likely to have ECG monitoring, and to receive PCI/CABG. They also were more likely to had good adherence to other medications at 6 months post discharge, including aspirin, clopidogrel, beta-blocker, and angiotensin-converting enzyme inhibitor/angiotensin receptor blocker **(**Table 1 **)**. The proportions of participants with 5, 10, 20, and ≥40 mg/d atorvastatin equivalent dose at discharge were 21, 33, 44, and 2%, respectively. The dosage levels were significantly higher in patients who were managed in tertiary hospitals compared to level-2 hospitals (*p* < 0.001)**(**Fig. 2 **)**.

**Table 1:** Baseline clinical characteristics according to the statin use adherence at six months after discharge

Baseline variables	Good adherence	Poor adherence	*P*-value
	(*n* = 9019)	(*n* = 3497)	
Studies, n(%)			
CPACS-1	1415(15.7)	512(14.6)	0.145
CPACS-2	7604(84.3)	2985(85.4)	
Male, n(%)	6380(70.7)	2390(68.3)	0.009
Age,mean(SD)	63.33(11.5)	63.34(11.8)	0.974
High School or higher, n(%)	3444(38.2)	1252(35.8)	0.013
Occupations, n(%)			
Employed	4605(51.1)	1767(50.5)	0.844
Not-employed	4214(46.7)	1654(47.3)	
Unknown	200(2.2)	76(2.2)	
Tertiary hospital, n(%)	5781(64.1)	1987(56.8)	<0.001
Having health insurance, n(%)	7308(81.0)	2700(77.2)	<0.001
Smoking, n(%)	2748(30.5)	1096(31.3)	0.343
Subtype of ACS, n(%)			
STEMI	3530(39.1)	1347(38.5)	0.104
Non-STEMI	1206(13.4)	427(12.2)	
Unstable angina	4283(47.5)	1723(49.3)	
CVD risk factors, n(%)			
Hypertension	6354(70.5)	2402(68.7)	0.053
Diabetes Mellitus	1892(21.0)	655(18.7)	0.005
Dyslipidemia^*^	4807(53.3)	1734(49.6)	<0.001
Smoking	2748(30.5)	1096(31.3)	0.343
Obesity	897(10.0)	321(9.2)	0.194
Family history of early CHD, n(%)	414(4.6)	171(4.9)	0.476
Higher heart rate(> = 100/min),n(%)	657(7.3)	277(7.9)	0.224
Continuous ECG monitoring, n(%)	6487(71.9)	2436(69.7)	0.012
Treatments during hospitalization, n(%)			
PCI/CABG	4746(52.6)	1334(38.2)	<0.001
Thrombolysis	608(6.7)	318(9.1)	<0.001
Statin before hospitalization	1681(18.6)	513(14.7)	<0.001
PCI/CABG in 0–6 months, n(%)	308(3.4)	131(3.8)	0.3663
Adherence of other medications in 0–6 months, n(%)			
Aspirin	8573(95.1)	2742(78.4)	<0.001
Clopidogrel	5962(66.1)	1414(40.4)	<0.001
Beta-blocker	6602(73.2)	1772(50.7)	<0.001
Angiotensin-converting enzyme inhibitor/Angiotensin receptor blocker	6312(70.0)	1493(42.7)	<0.001

*Dyslipidemia was defined as having a history of dyslipidemia or total serum cholesterol>=240mg/dL, or LDL-c>=160mg/dL, or triglyceride>=200mg/dL, or HDL-c<40mg/dL according to the guideline of China

### Adherence to statin and MACE

Among 12,516 patients with event-free survival to 6 months follow up, a total of 259 MACE occurred during the subsequent 6 months. The crude incidence rate of MACE events was statistically significantly lower in the good adherence group compared to the poor adherence group. After adjustment for co-variables, the association was attenuated, but remained statistically significant (Table 2).

**Table 2:** Clinical Outcomes at 7-to-12 Months between poor and good adherence group

Outcomes	Adherence groups	Number of events (%)	HR(95%CI)		
			Unadjusted	Age-sex-adjusted	Fully-adjusted ^a^
MACE	Poor adherence	95(2.72)	1(ref.)	1(ref.)	1(ref.)
	Good adherence	164(1.82)	0.66(0.51,0.85)	0.68(0.52,0.87)	0.73(0.56,0.97)
	*P* values	0.002	0.001	0.002	0.030
Total death	Poor adherence	56(1.60)	1(ref.)	1(ref.)	1(ref.)
	Good adherence	90(1.00)	0.62(0.44,0.86)	0.63(0.45,0.88)	0.71(0.49,1.04)
	*P* values	0.005	0.005	0.007	0.077
MI	Poor adherence	30(0.86)	1(ref.)	1(ref.)	1(ref.)
	Good adherence	66(0.73)	0.84(0.55,1.3)	0.85(0.55,1.3)	0.88(0.55,1.41)
	*P* values	0.468	0.444	0.447	0.6
Stroke	Poor adherence	21(0.60)	1(ref.)	1(ref.)	1(ref.)
	Good adherence	28(0.31)	0.51(0.29,0.9)	0.53(0.3,0.93)	0.56(0.3,1.02)
	*P* values	0.020	0.02	0.026	0.059

^a^Adjusted for studies (CPACS-1,-2), age, sex, education, occupations, have health insurance, hospital level, cardiovascular risk factors (hypertension, diabetes, dyslipidemia, obesity, smoking, and family history of early CHD), higher heart rate(> = 100/min), continuous ECG monitoring, subtype of ACS, thrombolysis, statin before hospitalization, PCI/CABG during hospitalization, PCI/CABG from 0- to 6- month after discharge, and adherence to clopidogrel, aspirin, beta-blocker, and ACEI/ARB

### Adherence to statin and components of MACEs

There were a total of 146, 96 and 49 patients resulted in death, myocardial infarction and stroke during follow up. The crude incidence rate of almost components of MACE was statistically significantly lower in the good adherence group compared to the poor adherence group, but after adjustment for co-variables, the associations were attenuated and not statistically significant (Table 2).

### Sub-group analyses

**Fig. 3:**
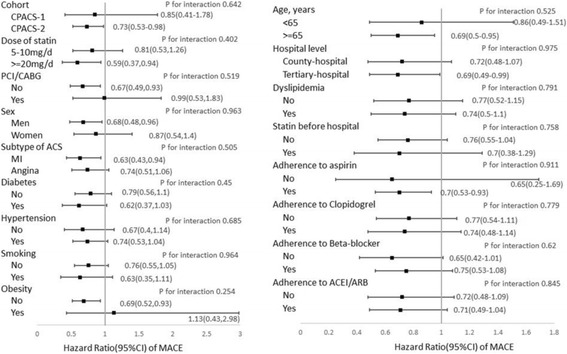
Hazard ratios of major adverse cardiovascular events (MACEs) for patients with good adherence compared to patients with poor adherence by cohorts (CPACS-1 and -2) and baseline characteristics. HRs were estimated with Cox model with adjustment for all other covariables shown in the fig. PCI = percutaneous coronary intervention. CABG = coronary artery bypass graft. ACEI = angiotensin converter enzyme inhibitor. ARB = angiotensin receptor blocker

All associations of adherence to statin with MACE were consistent between study cohorts and pre-defined subgroups of baseline characteristics including the sub-types of ACS (Fig. 3). There were not significant interactions between adherence to statin and other main exposure factors.

## Discussion

Our results demonstrate that good adherence to statin in the first 6 months after discharge is associated with subsequent lower incidence of MACE (including all-cause mortality, MI, and stroke) in a large unselected Chinese ACS patient population. This relationship was independent of other treatments which have been shown to be associated with outcomes in ACS such as receipt of coronary interventions, and adherence to antiplatelets, aspirin, beta-blocker, and angiotensin-converting enzyme inhibitor/angiotensin receptor blocker, as well as other known personal prognostic factors such as age, sex, education level, and cardiovascular risk factors and other hospital level characteristics. In addition, the effect of adherence was not modified by the characteristics of patients such as dose of statins, subtypes of ACS, gender, age, social economic status, etc.

It has been well established that good statin adherence is associated with reduced the risk of MACE and all-cause mortality in the primary prevention of coronary artery disease among healthy populations [15, 16]. The benefits associated with statin adherence in secondary prevention have only been reported among elderly AMI survivors for more than 1 year and three months^18^ and in known CVD (angina, myocardial infarction, heart failure, stroke/transient ischaemic attack and peripheral vascular disease as a whole) patients [19]. Such evidence among patients discharged with ACS is lacking and to the best of our knowledge, the present study is the first one to show good adherence to statin therapy during the first 6 months after discharge was significantly associated with lower risk of MACE in subsequent 6 months among ACS patients. Moreover, we found the relationship was not modified by statin dose, subtype of ACS, PCI/CABG, dyslipidemia, other clinical characteristics, and demographics, and was independent of good adherence to other evidence-based beneficial drugs, including antiplatelets, beta-blocker, and angiotensin-converting enzyme inhibitor/angiotensin receptor blocker at same time. Our findings suggest that adherence to statin is beneficial even in those ACS among whom about 50% did not have dyslipidemia and 54% had lower statin dose (<20 mg atorvastatin or equivalent).

Our findings highlight the importance to improve adherence to statin in the secondary prevention among ACS patients among Asian populations. We note that the level of good adherence to statin rate was only 72% at the 6 months after discharge in our study; considering the expected even lower adherence at 12 months as shown in CPACS-1 study [12], it was substantially lower than the adherence reported in Canadians [18]. Therefore, there is clearly room for improvement and ensuring good adherence to statins may translate into substantial clinical benefit with regard to MACE.

Our findings for each of the MACE components showed that the hazard ratio for patients with good adherence vs poor adherence was all less than 1.0 and borderline significant. We consider the results are coherent with the significant findings for the primary study outcome. Our ad-hoc power analysis indicated that the study power is 87% to detect the observed effect size on the primary outcome, MACE, 78% on total death, 13% on MI and 63% on stroke, with a = 0.05 among 12,500 study participants.

### Strengths

The participants were drawn from CPACS1 and CPACS2 which were well designed studies with high scientific quality standards [12, 20–22]. Firstly, we assessed the relationship of adherence to statin at 6 months after discharge with subsequent clinical outcomes during 7–12 months after excluding those patients with MI or stroke events in the first 6 months. This approach would avoid bias towards a result unfavorable to good adherence because the patients with recurrence of MI/stroke during the first 6 months were more likely to take statin at 6 month and hence be defined as adhere to statin in our study. Secondly, we controlled all available potential confounders including studies(CPACS-1,-2), age, sex, health insurance, social economic status, clinical characteristics, use statin before hospital, concomitant medicines and reperfusion therapies during hospitalization, and adherence to other evidence-based secondary prevention treatments for ACS in the first 6 months.

### Limitations

There are some limitations. First, we only defined good adherence as using statin without dosage reduction at 6 months, and poor adherence as using statin at discharge but dosage declined or discontinued usage at 6 months. Compared with common definition using the proportion of days covered (PDC) > =80% as good adherence, and 40–79%as poor adherence [23], we possibly misclassified some patients and thus under-estimated the strength of the associations observed. Second, as an observational study, we cannot assert causality due to the unmeasured and unknown confounding factors. Third, our data on adherence was self-reported, though a sample of study participants were randomly selected and central calls were made to confirm the quality of follow up data. Fourth, there was no independent adjudications of events in our study which make the diagnoses of MI/stroke might not be accurate. Finally, we only assessed the adherence at a single time point at relatively short interval of six-month post-discharge. Compared with longer-term adherence, the short-term adherence may have smaller magnitude of benefit as statins benefit has been more commonly acknowledged over at least 12–18 month therapy.

## Conclusion

In summary, this study found that ACS patients with good adherence to statin in the first 6 months after discharge had significantly lower risk of subsequent MACE in the following 7 to 12 months. Our study highlights the importance of adherence to cholesterol lowering therapy in prevention of MACE. Whilst the results are broadly similar to previous reports in Western populations, our study provide strong incentive with regard to relevance of statin adherence in reduction in MACE in the Chinese ACS patients.

## Additional file

Additional file 1: Table S1. Comparative Dose Efficacy of Statins on lipids (DOCX 15 kb)

## Abbreviations

ACEI: Angiotensin converter enzyme inhibitor
ACS: Acute coronary syndrome
ARB: Angiotensin receptor blocker
CABG: Coronary artery bypass graft
CI: Confidence interval
HR: Hazard ratio
MACEs: Major adverse cardiovascular events
MI: Myocardial infarction
PCI: Percutaneous coronary intervention
STEMI: ST segment elevation myocardial infarction
