# Supplementary material for: Six-month adherence to Statin use and subsequent risk of major adverse cardiovascular events (MACE) in patients discharged with acute coronary syndromes
Source: Lipids Health Dis. 2017 Aug 15;16:155. doi: 10.1186/s12944-017-0544-0 (PMC5558746; doi:10.1186/s12944-017-0544-0)
Supplement: Additional file 1: Table S1. — Comparative Dose Efficacy of Statins on lipids (DOCX 15 kb) [file 12944_2017_544_MOESM1_ESM.docx]

**Supplement A:** Comparative Dose Efficacy of Statins on lipids

| **Statin(mg)** | | | | |  | **Change of lipids (%)** | | | |
| --- | --- | --- | --- | --- | --- | --- | --- | --- | --- |
| **Atorvastatin** | **Simvastatin** | **Lovastatin** | **Pravastatin** | **Fluvastatin** |  | TC | LDL-C | HDL-C | TG |
| - | 10 | 20 | 20 | 40 |  | -22 | -27 | 4~8 | -(10~15) |
| 10 | 20 | 40 | 40 | 80 |  | -27 | -34 | 4~8 | -(10~20) |
| 20 | 40 | 80 |  |  |  | -32 | -41 | 4~8 | -(15~25) |
| 40 | 80 |  |  |  |  | -37 | -48 | 4~8 | -(20~30) |
| 80 |  |  |  |  |  | -42 | -55 | 4~8 | -(25~35) |

Source: Editor Committee of Chinese Guidelines on Prevention and Treatment of Dyslipidemia in Adults. Chinese Guidelines on Prevention and Treatment of Dyslipidemia in Adults in 2007." Chin J Cardiol 35, no. 5 (2007): 390-419.
